# Aptamers Against Live Targets: Is *In Vivo* SELEX Finally Coming to the Edge?

**DOI:** 10.1016/j.omtn.2020.05.025

**Published:** 2020-05-23

**Authors:** Mayte Sola, Ashwathi Puravankara Menon, Beatriz Moreno, Daniel Meraviglia-Crivelli, Mario Martínez Soldevilla, Fernando Cartón-García, Fernando Pastor

**Affiliations:** 1 Molecular Therapeutics Program, Center for Applied Medical Research, CIMA, University of Navarra, Pamplona 31008, Spain; 2 Instituto de Investigación Sanitaria de Navarra (IDISNA), Recinto de Complejo Hospitalario de Navarra, Pamplona 31008, Spain

## Abstract

Targeted therapeutics underwent a revolution with the entry of monoclonal antibodies in the medical toolkit. Oligonucleotide aptamers form another family of target agents that have been lagging behind in reaching the clinical arena in spite of their potential clinical translation. Some of the reasons for this might be related to the challenge in identifying aptamers with optimal *in vivo* specificity, and the nature of their pharmacokinetics. Aptamers usually show exquisite specificity, but they are also molecules that display dynamic structures subject to changing environments. Temperature, ion atmosphere, pH, and other variables are factors that could determine the affinity and specificity of aptamers. Thus, it is important to tune the aptamer selection process to the conditions in which you want your final aptamer to function; ideally, for *in vivo* applications, aptamers should be selected in an *in vivo*-like system or, ultimately, in a whole *in vivo* organism. In this review we recapitulate the implementations in systematic evolution of ligands by exponential enrichment (SELEX) to obtain aptamers with the best *in vivo* activity.

## Graphical Abstract

**Figure:**
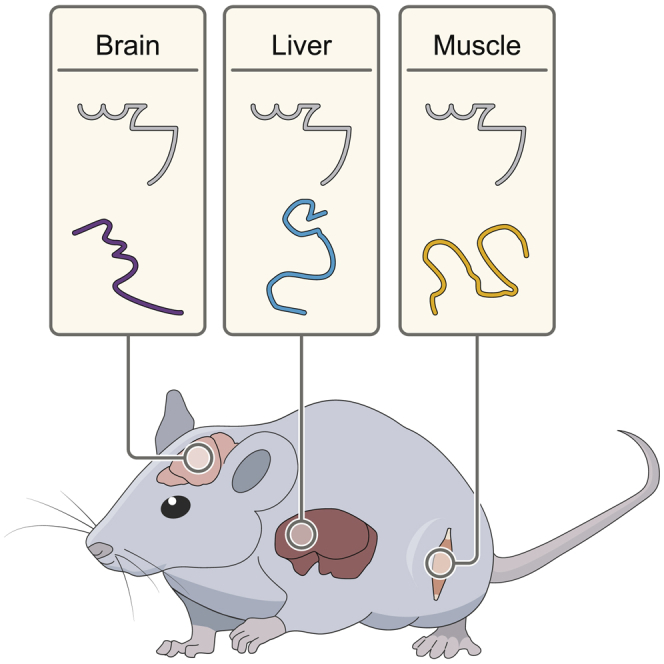

## Teaser

Oligonucleotide aptamers can function as targeting therapeutics agents for many biomedical applications. However, their full potential has not yet been translated into the clinical arena. Selection conditions for an aptamer can play an important role in determining its final *in vivo* activity. In this review we summarize some of the main modifications and improvements in the selection methods that allow one to obtain better aptamers for *in vivo* targeting.

## Main Text

Aptamers are synthetic single-stranded DNA or RNA molecules selected to bind to targets of diverse nature. They display several defined secondary motifs (e.g., loop, stem, or G-quadruplex) that allow them to adopt complex three-dimensional structures and confer these molecules the ability to recognize and bind targets with high affinity and specificity.^1^ Aptamers interact with their cognate targets with similar binding affinities to antibodies (dissociation constants in the low nanomolar/high picomolar range).^2^ In fact, aptamers have demonstrated high specificity, even discriminating between enantiomers^3^ or proteins that differ by only a few amino acid changes.^4^

Aptamers as oligonucleotides can be synthesized through straightforward phosphoramidite chemistry; consequently, they are known as “chemical antibodies.” Thus, similar to monoclonal antibodies (mAbs), aptamers can be developed for many different applications, either as diagnostic tools (biosensors) or as therapeutic agents.^2^^,^^5^, ^6^, ^7^, ^8^, ^9^ Different methods of aptamer selection have been described in the last few years, most of them based on an iterative selection process called systematic evolution of ligands by exponential enrichment (SELEX). In this review, we analyze in detail the main achievements that have been made in the optimization of this technology by focusing on the selection of aptamers against live targets, which will likely boost the discovery of more aptamers ideal for therapeutic applications.

Aptamers are highly clinically translatable and have a very favorable therapeutic potential. Many aptamers exhibit therapeutic effects themselves, but they can also be used as target agents to deliver different cargos to specific cells or tissues.^1^^,^^2^^,^^10^ Thanks to their small size, aptamers show high tissue penetration rates allowing efficient cell targeting and delivery of cargos such as proteins, small interfering RNAs (siRNAs), peptides, chemical drugs, microRNAs (miRNAs), or even other aptamers for specific targeting delivery *in vivo*^2^ (Table 1). Moreover, the chemical plasticity of aptamers allows for the addition of reporter groups that may be used as sensors in diagnosis.^5^^,^^6^

**Table 1:** List of Aptamers Selected for *In Vivo* Applications

Name	Aptamer Target	Selection Method	Application	Sequence	Reference
N55	inflamed endothelial cells	stimulus-response cell-SELEX (SRC-SELEX)	atherosclerosis plaque detection	5′-ATACCAGCTTATTCAATTCC CAAATTGCCACCACTTACAG CATGATAACATACTACATCTT TTCATCAAGATAGTAAGTGCAATCT-3′	Ji et al.^24^
GBI-10	tenascin-C	cell-SELEX	diagnosis and treatment delivery in several types of tumors	5′-GCCTGTTGTGAGCCTCCTCCC AGAGGGAAGACTTTAGGTTCGGTT CACGTCCCGCTTATTCTTGTCTCCC-3′	Daniels et al.^32^
SQ-2	ALPPL-2	cell-SELEX	identification of novel biomarkers for PDAC early diagnosis	5′-AUACCAGCUUAUUCAAUUGCC UGAAAAGCUAUCGCCCAAUU CGCAGUGAUAUCCUUUAAGAUAG UAAGUGCAAUCU-3′	Dua et al.^52^
NK2	H37Rv strain of MTB	cell-SELEX	tuberculosis treatment	5′-GCGGGATCCTATGACGCATTGACCCA CAACACACTACTGTCGCTCGGTTCGA ACTTCGTGCGACTGTTCCCTATAG TGAGTCGTATTAGAATTCCGC-3′	Chen et al.^61^
G-3	CCR5	cell-SELEX and HTS combination	HIV infection blockade	5′-UAAUACGACUCACUAUAGGGAGG ACGAUGCGGGCCUUCGUUUGUU UCGUCCAUCGGGCGAGUCGUCUG-3′	Zhou et al.^72^
P30-10-16	HA of influenza B virus	*in vitro* SELEX	influenza B virus infection blockade	5′-GGGAGAAUUCCGACCAGAAGAUUAUG CAGUUUCAUUAUAUCAUACACCAA CCUUUCCUCUCUCCUUCCUCUUC-3′	Gopinath et al.^73^
A07	TGFBR3	TECS-SELEX	inhibition of the interaction between TGFBR3 and TGF-β2 *in vitro*	5′-GGGCCAGGCAGCGAGAGAUAAGCAGAA GAAGUAUGUGACCAUGCUCCAGAGA GCAACUUCACAUGCGUAGCCAAAC CGACCACACGCGUCCGAGA-3′	Ohuchi et al.^77^
MRP1Apt (3)	MRP1	peptide-SELEX and cell-SELEX	tumor cell targeting for treatment delivery	5′-GGGAGAGGGAGAAUAGUCAACAAAUCGU UUGGGGCGACUUCUCCUUCCUUUCUCCC UUCUCCC-3′	Soldevilla et al.^10^
Apt02	ITGAV	Icell-SELEX	discovery of ligands for pharmaceutically challenging targets	5′-GGGAUCCGCAUCUAGAGUACUCCUCAG GCUUCAAUGCUUACGCAAUCCUGGGG GUCGAUGAACGUCUACUGAAGCUAUCA-3′	Takahashi et al.^78^
Sgc8	PTK7	cell-internalized SELEX	targeting ALL cells for drug delivery	5′-ATCTAACTGCTGCGCCGCCGGGAA AATACTGTACGGTTAGA-3′	Xiao et al.^85^
A1	HER2	cell-internalized SELEX	targeting HER2-positive breast cancer cells	5′-GGGAGGACGAUGCGGGACUGUACG GGGCUCUGUGCAGACGACUCGCCCGA CAGACGACTCGCTGAGGATC GAGA-3′	Thiel et al.^82^
J7	CD3ε	LIGS	expansion of T cell repertoire	5′-AAGGAGCAGCGTGGAGGATATCGGTAA GGGTCGGGGATGCTACAACTGTTTAAAC GACCCGTCCATTAGGGTGTGTCGTCGTGGT-3′	Zumrut et al.^90^
RNA 14-16	p68 oncogenic helicase	whole-organism *in vivo* SELEX	localization of metastasis in the liver	5′-GGGAGGACGATGCGGCAGUGCCCAA CCGGAACAACAACCACCGGCGGCU CCUGCU-3′	Mi et al.^98^
PB	activated endothelial cells	whole-organism *in vivo* SELEX	identification of bone metastases in prostate cancer	5′-CTCTATTGATGCCTGCGTGCGTGC TTGTAG-3′	Chen et al.^102^
GL21.T	Axl	cell-SELEX	treatment of Axl-dependent cancers	5′-AUGAUCAAUCGCCUCAAUUCGACAG GAGGCUCAC-3′	Cerchia et al.^37^

They also provide several substantial benefits compared to conventional therapeutics such as antibodies. First, the existence of antidotes—either in the form of an oligonucleotide with a sequence complementary to the aptamer, or a universal antidote based on positively charged proteins or polymers—capable of binding to the aptamer and disrupting its structure and function *in vivo* is beneficial.^11^^,^^12^ The access to this sort of antidotes is amenable to safer drug design and allows aptamers to perhaps represent a unique class of therapeutic agents that have an important safety advantage over other therapeutic classes of molecules. Second, aptamers are chemically synthesizable, which facilitates their large-scale production in good manufacturing practice (GMP) grade and relatively lower cost of production. Finally, their small size confers them lower antigenicity, which decreases the chances of inducing unwanted humoral T cell-dependent immune responses.

However, despite their therapeutic potential and success in some pre-clinical models, aptamers are still not major players in the clinical trial pipeline. Several reasons might contribute to this. First, they compete with conventionally accepted and vetted mAbs in the same therapeutic niche. Second, they show poorer pharmacokinetics than antibodies and require modifications to improve their half-life *in vivo*. Third, a large phase clinical trial that held great expectations using an anticoagulant aptamer (RADAR phase 2b clinical trial) showed toxicity in only a small fraction of patients. Unfortunately, this outcome likely discouraged the launch of other clinical trials using this technology. It is important to highlight that the toxicity observed in this clinical trial turned out to be associated with the pre-existence of antibodies against PEG (polyethylene glycol), a modification included in the anti-coagulant aptamer to enhance its half-life in serum.^13^ There are some clinical trials ongoing with aptamers,^9^ and the success of any of them will hopefully foster and expedite the initiation of others. Lastly, and most importantly, there are several important challenges that need to be overcome to identify aptamers that function optimally not just *in vitro*, but also *in vivo*. Through this review, we hope to survey and address some of these challenges in developing aptamers to function *in vivo* and discuss how SELEX against a live target will bridge this clinical gap.

### SELEX

**Figure 1:**
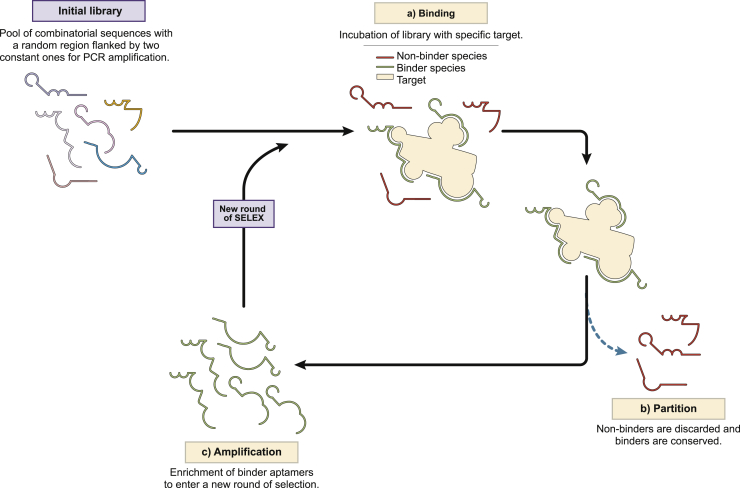
Depiction of the Steps of Conventional Systematic Evolution of Ligands by Exponential Enrichment (SELEX)

SELEX is an iterative selection process where an oligonucleotide aptamer library is exposed to the desired target in various repetitive cycles. The protocol for the *in vitro* selection of aptamers (SELEX) was developed in 1990 by Ellington and Szostak^14^ and by Tuerk and Gold,^15^ who demonstrated the capacity of aptamers to target a large variety of molecules (Figure 1). Every round of SELEX consists of three main steps: (1) binding, (2) partition, and (3) amplification. The initial SELEX library consists of a pool of randomized, combinatorial oligonucleotide sequences, with a random region flanked by two fixed constant regions that are used for primer annealing and required for amplification by polymerase chain reaction (PCR). Briefly, during the binding step, the aptamer library is incubated with the target molecule, and aptamer species that bind to the target are pulled down and isolated from the sequences that are weakly bound or do not interact (partition). During the amplification step the selected oligonucleotides are amplified by PCR (in case of DNA) or by reverse transcriptase PCR (in the case of RNA) to enrich the library. From the 1990s to date, SELEX has evolved significantly;^16^ new methods in aptamer synthesis, technical equipment, and analysis have increased the efficiency of the method. During the last few years several chemical modifications of the sugar (pentose)-phosphate backbone and bases have been reported to change the nature of the aptamers to improve their affinities, stability, and pharmacokinetics.^17^

Furthermore, conventional SELEX needs several weeks to be completed but, thanks to the incorporation of the high-throughput sequencing (HTS) platform, the process can be expedited to a few weeks by reducing the number of cycles for each SELEX. HTS provides millions of reads per sample, which allows the identification of enriched aptamer species in early rounds of selection. Information retrieved from HTS serves to (1) monitor the enrichment of the sequences as the selection progresses;^18^ (2) reduce the number of rounds and bias associated with PCR and transcription;^19^ (3) identify higher number of aptamer clusters;^18^ and (4) recognize conserved motifs that might be involved or required for binding to the cognate target.^18^^,^^20^^,^^21^ Despite the efficiency of the SELEX procedure, aptamers selected through conventional SELEX using a recombinant protein target may fail to recognize the native target expressed on the cell surface. Aptamers can discriminate between closely related molecules; therefore, modifications on target recombinant proteins (e.g., linker, tags, glycosylation, misfolding) used for selection may hinder subsequent binding to native protein targets *in vivo*. Furthermore, the target must be well characterized and purified; protein targets for SELEX are chosen with a tag (e.g., Fc, His) to favor the partition step, but this tag includes extra amino acids that can bias and confound the aptamer selection. In a quest to overcome this limitation, the use of targets expressed in their native state on live organisms has been implemented. This type of SELEX is facilitated by various biological entities, starting from infectious agents such as viruses, bacteria, or parasites, to cell lines or mammalian-derived primary cells, to *ex vivo*-engineered tissues and even whole living animals. Hence, in this review, we use the term “cell-SELEX” when live cells are designated as targets, “tissue-SELEX” when selection is performed against *ex vivo* tissue-derived structures or tissue-mimicking structures, and, finally, “whole animal*-*SELEX” when the selection procedure is performed with live animals.

### Cell-SELEX

Cell-SELEX is based on using intact, living cells to obtain aptamers that specifically bind to one or multiple targets on the surface membrane of the same cellular type. The main feature of cell-SELEX is that it improves the chances of selecting aptamers binding to the targets on the cell surface in their native form. Moreover, using cells for SELEX has the advantage that no previous knowledge is required about the target molecule; aptamers can potentially interact with any component of the cell membrane, encompassing the entire range of membrane proteins, lipids, and polysaccharides. Through this strategy, aptamer selection is driven toward selecting the most effective species that are assured binders to the target cell of choice. Cell-SELEX may also be utilized as a method to identify new specific biomarkers on the surface of a cell subtype. Aptamer-binding targets can be characterized afterward by co-precipitation and mass spectrometry analysis^22^ to resolve these markers on the cell.

**Figure 2:**
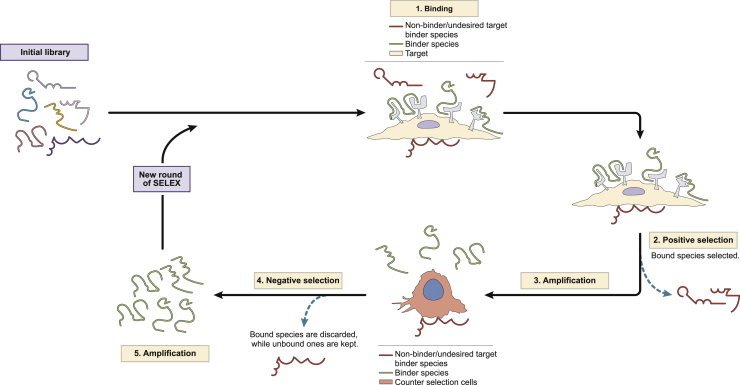
Sequential Steps in Cell-SELEX

The cell-SELEX protocol is divided into different steps similar to regular SELEX (Figure 2). Counter-selection rounds are extensively used in most variants of SELEX to increase the specificity of selection by removing aptamers that do not bind to cognate ligands. In cell-SELEX, it is crucial to include a counter-selection step, as there are many potential non-desirable targets that can mislead selection. For counter-selection, the parental cell line or a similar one in terms of ontogenicity, morphology, and phenotype should be used. In the case of selection against a type of tumor, the cell line chosen for negative selection should be the non-transformed correspondent of the tumor cell line, as this guarantees that the aptamers selected are able to differentiate between malignant cells and healthy ones.

Cell-SELEX has been extensively used to identify aptamers against different cellular types involved in several diseases, such as synovium inflammation,^23^ atherosclerosis,^24^ obesity,^25^ hyperglycemia in patients with diabetes mellitus,^26^ cancer,^27^ and microorganisms involved in infectious diseases,^28^ among others. Cell-SELEX can be performed against almost any type of cell line, primary derived cell, bacterium, parasite, or virus.

#### Cell-SELEX Targeting Unrevealed Receptors

As mentioned earlier, a key characteristic of cell-SELEX is that no prior knowledge about the nature of the target is required for the selection of a targeting aptamer. Moreover, the technique is so powerful that it can even generate aptamers that are able to distinguish between subpopulations of the same cell type that cannot be characterized by conventionally available methods. This has enabled cell-SELEX to be a successful tool for the discovery new biomarkers^29^^,^^30^ and for targeted cell therapy.^7^^,^^27^^,^^31^

One of the first cell-SELEX procedures was performed in 2003 with a glioblastoma-derived cell line (U251).^32^ Twenty-one rounds of cell-SELEX were performed, and the sequences obtained were cloned and sequenced. The analysis of these sequences led to the identification of a predominant aptamer, GBI-10, which showed binding to U251 glioblastoma cells.^32^ In order to identify the aptamer-protein target, the proteins were isolated through an affinity purification procedure using biotinylated aptamers immobilized on streptavidin-coated magnetic beads. The pulled-down, aptamer-protein-bound fraction was separated and further analyzed via mass spectrometry. The GBI-10 aptamer was resolved as a binder to tenascin-C, an extracellular protein involved in pivotal pathways such as embryogenesis and oncogenesis.^33^ Subsequently, the GBI-10 aptamer was used in different therapeutic preclinical approaches, such as targeting adenovirus (Ad) to the glioblastoma U251 cell line, enhancing the transduction efficiency of GBI-10-modified Ad.^34^ The GBI-10 aptamer was also used to guide gadolinium-loaded liposomes as a carrier of contrast agent in magnetic resonance imaging (MRI) for diagnosis and tumor therapy.^35^ Recently, the GBI-10 aptamer has also been used to guide nanoparticles for therapy in pancreatic ductal adenocarcinoma (PDAC) that grew in an extracellular matrix (ECM) enriched in tenascin-C protein.^36^ In this study, nanoparticles of camptothecin dimeric prodrug (CPTD) were encapsulated with cell-penetrating peptide (CPP) in order to increase the cellular drug uptake. The conjugation of GBI-10 aptamer to the drug complex allowed for improved tumor-selective targeting, increasing antitumor response *in vivo* and reducing systemic toxicity.^36^

In similar lines, Cerchia et al.^37^ also developed a GL21.T aptamer that specifically binds the U87MG glioma cell line and that was further characterized to bind the extracellular domain of the AXL receptor tyrosine kinase (RTK). Importantly, transmembrane receptor tyrosine kinases play crucial roles in cancer cell proliferation, survival, migration, and differentiation, constituting important targets for therapeutic aptamers.^38^ Thus, GL21.T has been shown to inhibit cell migration, invasion, and *in vivo* lung tumor formation in mice xenografts by blocking Erk and Akt phosphorylation,^37^ and similar results have also been observed in ovarian cancer models.^39^ Interestingly, the therapeutic potential of GL21.T was improved by developing an aptamer-miRNA complex (AmiC) composed of the GL21.T aptamer and miR-137, downregulated in lung cancer and involved in cell survival and proliferation. After non-small cell lung cancer (NSCLC) treatment, they observed a combination of the antagonist effects of GL21.T aptamer binding the Axl oncogenic receptor, together with increased miRNA cellular levels and downregulation of miRNA targets. These effects significantly impaired NSCLC migration and proliferation.^40^ The generation of AsiC chimeras (aptamer-linked siRNAs) has also been reported using GL21.T aptamer conjugated to let-7g miRNA, which was able to silence let-7g target genes, thereby reducing tumor growth in a xenograft model of lung adenocarcinoma.^41^

Cell-SELEX has been used to identify aptamers against a plethora of cancer cell lines to spearhead the innovation of potential therapeutic agents and for the discovery of new biomarkers. For instance, several new molecular targets for cancer stem cells (CSCs) were identified using cell-SELEX.^29^^,^^42^ CSCs have been underscored as major players involved in metastasis promotion and chemotherapy resistance. They develop mechanisms to avoid drug and radiation damage and are not very abundant in the primary tumor. The absence of specific biomarkers for CSCs impedes the development of effective therapies against them.^43^

Cell-SELEX was also performed against cell lines of PDAC,^44^ gliosarcoma,^45^ and liver,^46^^,^^47^ colorectal,^48^ lung,^30^^,^^49^ prostate,^50^ and breast cancer,^51^ among others. With the aim of finding a new biomarker for the diagnosis of PDAC, Dua et al.^52^ carried out cell-SELEX against two target human pancreatic cell lines, Panc-1 and Capan-1, using normal human pancreatic ductal cells for negative selection. As a consequence, they were able to isolate the SQ-2 aptamer, whose ligand was identified as alkaline phosphatase placental-like 2 (ALPPL-2), an oncofetal protein that is also overexpressed in testicular cancers.^53^^,^^54^ Given the expression of ALPPL-2 in PDAC, the discovery of the SQ-2 aptamer has thus allowed for a novel method for early-stage detection of this aggressive type of cancer.

As we have previously explained that every molecule on the cell surface could be a potential target for identifying aptamers via SELEX. Suitably, several aptamers targeting different receptors expressed on the target cell can be isolated simultaneously in a single cell-SELEX. For instance, seven different aptamers against various targets were identified from a single cell-SELEX performed against metastatic colorectal cancer (CRC) LoVo cells.^48^ These aptamers were later used as carriers for the specific delivery of doxorubicin to the target cells and, consequently, were able to reduce the off-target effects of doxorubicin therapy significantly.

#### Cell-SELEX Targeting Pathogens

Cell-SELEX can involve different kinds of cells, including bacteria and parasites, as well as viruses, even if the latter might not be considered as cells. The microbiology field has also taken advantage of this, and several works have been published using cell-SELEX to select aptamers that target various pathogens.^28^^,^^55^

The malaria pathogen *Trypanosome* undergoes transient modifications on its surface to avoid recognition by the immune system.^56^ The cell-SELEX technique, successfully exploited, allowed for the identification of aptamers that recognize several strains of *Trypanosome* and some of these modifications.^57^, ^58^, ^59^ Aptamers against other parasites, such as *Cryptosporidium*, have also been developed, making detection in food possible.^60^

One of the first cell-SELEXes performed against whole bacteria was carried out by Chen et al.^61^ in 2007. The selected aptamer NK2 bound to H37Rv strain of *Mycobacterium tuberculosis* (Mtb) with high affinity and specificity. Aptamer binding to H37Rv increased interferon-γ (IFN-γ) production by CD4^+^ T cells. Mice challenged with H37Rv and treated with a single injection of the aptamer showed prolonged survival rates, suggesting that aptamer NK2 can be used as an anti-mycobacterial drug. Likewise, cell-SELEX has also been used to identify aptamers against a wide variety of bacteria, ranging from *Campylobacter*^62^ to *Salmonella*,^63^^,^^64^ *Escherichia coli*,^65^ *Haemophilus*,^66^ *Neisseria*,^67^ *Vibrio*,^68^ and others.^69^

This opens up the possibility of developing antagonistic aptamers against infectious microorganisms that block their ability to invade their host. Of particular interest are the Rous sarcoma virus (RSV) and human immunodeficiency virus (HIV), both members of the Retroviridae family. These enveloped viruses express essential glycoproteins on the surface that allow for host-cell recognition and therefore infection. One therapeutic approach to preclude viral infection is the development of aptamers that block viral cell penetration.^70^ Pan et al.^70^ were able to select a pool of RNA and RNA analogs that neutralized RSV by interacting directly with the virus while leaving the host cells unaffected.

The use of aptamers for HIV diagnosis and therapy has also been actively pursued.^71^ C-C chemokine receptor type 5 (CCR5) is a co-receptor required for HIV infection, and deficiencies in CCR5 expression have been associated with resistance to HIV infection. Zhou et al.^72^ combined cell-SELEX and HTS to target HIV-1-susceptible cells and block CCR5, thus preventing HIV infectivity. They selected a candidate aptamer G-3 that was able to bind and be internalized by human CCR5-expressing cells, thus competitively inhibiting and preventing HIV binding and consequent infection.

Similarly, Gopinath et al.^73^ described a hemagglutinin (HA)-blocking aptamer, P30-10-16. HA is a viral antigen necessary for influenza virus infection. The P30-10-16 aptamer bound with high affinity to HA (15-fold-higher than the corresponding mAb) and inhibited the virus membrane fusion with host cells.

Viruses and other intracellular pathogens can selectively induce modifications on the cell surface of the infected cells. Based on this knowledge, cell-SELEX can be used advantageously to isolate aptamers that can recognize only infected cells. This type of selection has already been carried out with vaccinia virus-infected cells,^74^ rabies virus,^75^ papillomavirus,^76^ as well as with Mtb bacteria.^61^

#### Cell-SELEX against Specific Surface Receptors

In many cases, cell-SELEX can be used to isolate target-specific aptamers against known ligands expressed on the cells. Target expressed on cell surface SELEX (TECS-SELEX) is a method of SELEX in which selection is performed against a chosen target using cells that artificially express that target protein on the cell surface.^77^ In Ohuchi et al.^77^ TECS-SELEX was performed using a CHO-K1 cell line genetically modified to ectopically express human transforming growth factor β (TGF-β) type III receptor (TGFBR3) on the cell surface. Parental non-modified CHO-K1 cells were used for counter-selection in order to lead the selection toward the desirable target (TGFBR3).

In the same way, Soldevilla et al.^10^ developed a protocol that combined peptide-SELEX and cell-SELEX to isolate an aptamer that binds to a peptide from the extracellular domain of MRP1, a 17-transmembrane protein. After 10 rounds of peptide-SELEX, they performed a final round of cell-SELEX with H69AR, a tumor cell line with high expression of MRP1, and the parental cell line H69 as counter-selection. This MRP1 aptamer was later used for targeting other immunostimulatory aptamers to the tumors for cancer immunotherapy in preclinical tumor-bearing mouse models.^10^

Takahashi et al.^78^ introduced a new modification of cell-SELEX called “isogenic cell-SELEX” (Icell-SELEX). In Icell-SELEX, cell-surface target proteins can be overexpressed for positive selection and downregulated for counter-selection, using RNA interference (RNAi)-mediated silencing. Using this method, an anti-integrin alpha V (ITGAV) aptamer was obtained. This approach endows SELEX screening with the possibility of selecting aptamers against low-expression receptors or targets that are broadly expressed by fostering a wider window of expression between the target cell line and the parental one.

### Technical Variants of Cell-SELEX

#### FACS-SELEX

One of the most critical steps in SELEX is the partition of unbound aptamers after selection. Fluorescence-activated cell sorting (FACS) is a suitable method for cell separation that can be used in this partition step. FACS allows for the identification of complex cellular subpopulations depending on their markers. In FACS-SELEX, one or more fluorescently labeled antibodies with specificities for desired subpopulations can be used for the enriched separation of the aptamer-bound fraction by FACS, allowing for increased specificity of selection. Since aptamers bind non-specifically to dead cells, cell-SELEX is susceptible to triggering non-specific bindings of aptamers to dead cells, and skewing or biasing the selection process. This caveat is overcome via FACS-SELEX, which permits differentiating dead versus viable cells using dead cell-specific markers.

Raddatz et al.^79^ used FACS-SELEX to select aptamers that bind to Burkitt’s lymphoma cells with a vital cellular phenotype. After several rounds of FACS-SELEX, two aptamers were identified as binders to Burkitt’s lymphoma cells.

#### Cell-Internalized SELEX

The charge and size of oligonucleotide aptamers might preclude their uptake and cytosolic translocation *in vivo*.^80^ For intracellular delivery of a therapeutic payload (e.g., toxin, drug, siRNA) the aptamer needs to target a receptor that is taken up by the target cell. To that end, cell-SELEX can be modified in order to select cell-internalizing aptamers by eliminating all of the aptamers that remain bound to the extracellular proteins on the cell surface. Different approaches have been undertaken to strip all of the extracellular aptamers after previous incubation with the aptamer library, some of which include using a high-salt wash, trypsinization, and RNase treatment during the partition step. High-salt washes induce a conformational change on the extracellular-bound aptamers, thereby liberating them from the cell surface. HER2 aptamers and aptamers against myoblasts were selected using this approach.^81^^,^^82^ Trypsinization can also be used to remove extracellular proteins and, therefore, the bound-aptamer fraction that has not been internalized.^81^^,^^83^^,^^84^ Sgc8 aptamer was selected to bind to human protein tyrosine kinase-7 (PTK7), which is a specific biomarker of acute lymphoblastic leukemia (ALL) T cells.^85^ Other internalizing aptamers against breast cancer cell lines were identified using this cell-SELEX variant.^84^ Lastly, RNase treatment has also been successfully used to remove non-internalized aptamers during the selection, allowing for the identification of cell-internalizing aptamers against pancreatic cancer cell lines.^83^

#### On-Chip Cell SELEX

Cell-SELEX is still a long and cumbersome technique, and thus improvements in this technology have been actively pursued. Microfluidic systems have been adapted in order to decrease the time and labor that multiple SELEX rounds imply through an automatized process. The on-chip cell-SELEX process is based on a microfluidic system capable of integrating all SELEX steps on the same chip. It consists of a sealed microfluidic chip manufactured with polydimethylsiloxane (PDMS) and glass substrate, designed with several chambers for the different steps of the process. The randomized, variable DNA library is incubated with target cells to isolate the bound aptamers, which are mixed with the control cells later. Then, unbound aptamers are transported to an amplification chamber. The amplified DNA constitutes the successive rounds of the cell-SELEX process. On-chip cell-SELEX allows for a viable, automated system that reduces the time of each SELEX round. Several aptamers have been selected by on-chip cell-SELEX for use in the early detection of ovarian cancer (OvCa),^86^ CRC and CRC stem cells (CR-CSCs),^87^ as well as cholangiocarcinoma cells.^88^

#### Ligand-Guided Cell-SELEX

Ligand-guided cell-SELEX (LGCS) is a variant of cell-SELEX that allows guiding aptamer selection toward a specific target of the cell surface using cognate ligands, such as antibodies, to that target. First, the aptamer library is incubated with target cells expressing the chosen receptor. Then, the receptor-binding antibody or ligand to the target receptor is added to selectively displace aptamers attached to the same binding pocket as the ligand. These detached aptamer species are collected and PCR amplified to constitute the library that is used to repeat the next rounds of selection. This approach works well to identify aptamers against specific epitopes that are shared by an antibody. A caveat of this type of selection, however, is that the aptamer affinity is unlikely to surpass the actual ligand-receptor affinity.

In another study, Ulrich et al.^59^ selected aptamers against the Chagas disease-causing parasite *Trypanosoma cruzi*. Using LGCS, they successfully identified aptamers that bound to parasitic proteins essential for host-cell adhesion and invasion. The aptamer library was incubated with the parasites, and unbound species were removed by centrifugation. Next, the parasite-bound aptamer fraction was incubated with a solution containing ligands known to be involved in parasite-host cell adhesion, such as laminin, fibronectin, heparan sulfate, and thrombospondin. Aptamers competing for the same binding site as the natural ligands were displaced and collected by an additional centrifugation step and used for the next rounds of selection, thus resolving an aptamer that could partially block invasion of epithelial monkey kidney host cells *in vitro*.

Similarly, Zumrut et al.^89^ performed cell-SELEX against an immunoglobulin M expressed on the cell membrane (mIgM) of Ramos cells of Burkitt’s lymphoma. An antibody against mIgM was used as a ligand to displace the mIgM-bound aptamers. The same group, in a later study, optimized this technique to obtain aptamers with high affinity and specificity against the CD3ε subunit of the TCR complex using the CD3 antibody as the competing antibody in ligand-guided selection (LIGS). They selected DNA aptamers against TCR-CD3ε with affinities from 3 to 300 nM^90^ that could bind to TCR-CD3 in its native conformation.

#### Toggle-Cell-SELEX

Toggle-cell-SELEX is aimed at selecting aptamers with cell-to-cell cross-reactivity by aptamers that either bind to a common receptor or to a highly homologous receptor expressed among the different target cells used in the selection. Toggle-cell-SELEX is usually conducted by swapping each selection round against a different chosen target cell line. Hence, at the end of the selection the identified aptamers might recognize all or several target cell lines used in the selection.

A sequential toggle-cell-SELEX was performed by Song et al.,^91^ who selected aptamers against both Gram-positive and Gram-negative bacteria. In this study, different bacteria, including *Escherichia coli*, *Enterobacter aerogenes*, *Klebsiella pneumoniae*, *Citrobacter freundii*, *Bacillus subtilis*, and *Staphylococcus epidermidis*, were used in each round of selection.

Another example of toggle-cell-SELEX was performed to select an aptamer with cross-reactivity between human and mouse endothelial cells.^92^ The procedure began with the first round of cell-SELEX against both cell lines separately, corresponding to brain microvascular endothelial mouse (bEND3) and human (hCMEC/D3) cell lines. The selection continued with equimolar quantities of aptamers obtained in the first round of both cellular types, and the following rounds were performed alternating the two cell lines. Toggle-cell-SELEX could be used to identify aptamers that can cross-react among different species as it was initially described with proteins.^93^ Another possible application is to identify aptamers that recognize a set of receptors that belong to the same family (e.g., tumor necrosis factor receptor [TNFR]).

### Tissue-SELEX

Cell-SELEX offers numerous advantages over conventional SELEX; however, some aptamers have been selected by cell-SELEX with high affinity that later failed in *in vivo* studies. Aptamers are highly specific molecules, so any modification in the environment of the aptamer target can reduce their affinity. Therefore, one of the most important aspects in cell-SELEX is the condition and nature of the cells used for selection, which must always be similar to physiological conditions *in vivo*, which is not always easy to achieve. Furthermore, tissues and organs in a living organism are formed by different types of cells whose complexity, heterogeneity, and morphological structure are difficult to recapitulate in cell culture. Consequently, even aptamers selected through cell-SELEX could fail in target recognition *in vivo*. In the quest to improve aptamer binding in living complex organisms, selection via tissue-SELEX, which utilizes whole tissue as the target for binding, presents itself as a favorable option.

Aptamers selected by cell-SELEX to bind to smooth muscle cells were screened in whole arteries *in vivo* to identify aptamers that bind to the arterial walls.^94^^,^^95^ Tissue-SELEX was also performed against tumor sections from ductal carcinomas identifying hnRNP A1 as a target.^96^ One variant of tissue-SELEX is the Morph-X-Select, morphology-based tissue aptamer selection. Wang et al.^97^ used tissue sections from patients to identify high-affinity aptamers and also the target proteins associated with ovarian cancer. The initial DNA library had been modified with thiophosphate substitutions at selected positions to increase nuclease resistance and targeting. Furthermore, they used image laser microdissection (LMD) to dissect exclusively the regions of interest bound with the thioaptamer. Finally, they were able to identify and characterize a candidate, V5, vimentin-specific sequence with specific binding to tumor vasculature of human ovarian tissue and human microvascular endothelial cells.^97^

### Whole-Organism *In Vivo* SELEX

**Figure 3:**
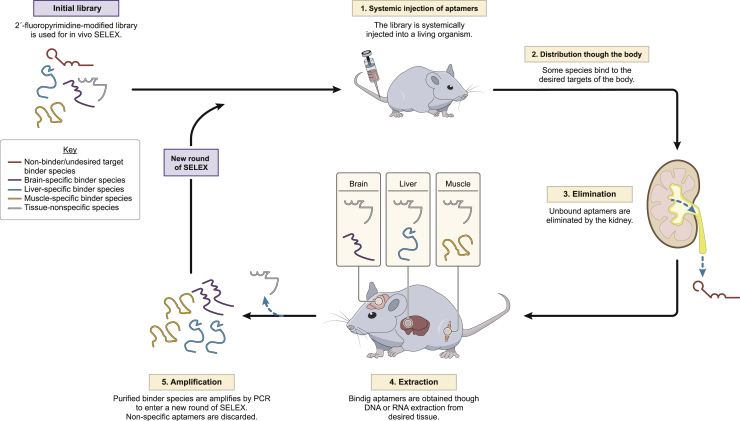
Whole-Organism *In Vivo* SELEX

Whole-organism *in vivo* SELEX starts with a random library of 2′-fluoropyrimidine-modified RNA or DNA aptamers that are injected systemically into a living animal or plant and allowed to circulate through the whole organism (Figure 3). During exposition time, aptamers are distributed throughout the body, with certain aptamer species binding to the desirable target tissues and unbound aptamer species being eliminated by the kidney. Afterward, the target organ/tissue must be removed from the organism for RNA or DNA extraction. The isolated aptamers are amplified from the target organ/tissue via PCR with specific primers that match the flanked constant regions of the starting library in order to amplify only aptamers accumulated in the target tissue. As in conventional SELEX, RNA libraries need to be retrotranscribed and *in vitro* transcribed, before and after amplification, respectively. The new pool of aptamers acquired from the previous round is re-injected in the same organism and the same process is followed for several rounds, enriching the highest affinity binders to the target tissue or organ of interest (Figure 3). Finally, aptamers that have been enriched in the library can be identified by HTS.

In conventional SELEX using purified recombinant protein as well as in cell-SELEX, a negative selection process is usually carried out to remove non-specific binding molecules in a counter-selection step. In whole-organism *in vivo* SELEX, the counter-selection is intrinsic to the method. Other non-targeted tissues should be analyzed using next-generation sequencing to withdraw non-specific aptamers. As the aptamer library re-circulates through the body, non-binding aptamers are eliminated via the kidneys, and abundant non-target tissue in other parts of the body serves as a control for aptamer specificity. Aptamer species that are contained in the desirable organ but also in other analyzed tissues are to be withdrawn as non-specific aptamers (Figure 3).

When the SELEX procedure is carried out with a living animal, RNA molecules circulate through the organism and may potentially bind to targets expressed on the tissue of interest. Therefore, there is no prior knowledge of the protein targets that will dominate the aptamer selection, so this approach could also be used to identify new tissue-specific markers combining aptamer-protein precipitation and mass spectrometry. In the case of cancer, this strategy is particularly attractive given that the tumor microenvironment is highly complex, heterogeneous, and multifaceted, making tissue-SELEX and whole-organism *in vivo* SELEX effective tools to select aptamers that are physiologically representative and bind to tumor tissue

Mi et al.^98^ performed whole-organism *in vivo* SELEX in a model of intrahepatic CRC metastases. After 14 rounds of *in vivo* selection collecting the aptamer fraction that bound to liver metastases, they identified an aptamer that bound to and blocked the activity of the oncogenic helicase p68 that turned out to be upregulated in the tumor.

Similarly, whole-organism *in vivo* SELEX can be used as a novel tool to target human cancer cells in living tissue implanted in animals as xenografts. This is a more precise method to identify a specific aptamer against human cancer cell lines *in vivo*.^99^ Two different cell lines were implanted separately for the intrahepatic xenografts obtained from human patients with colorectal liver metastases to perform the *in vivo* SELEX. As a result of that study, Mi et al.^99^ identified an anti-DHX9 tumor aptamer. DHX9 is RNA helicase A involved in transcriptional regulation, which was previously described to be overexpressed in CRC.^100^

Wang et al.^101^ tried to target NSCLC using whole-organism *in vivo* SELEX with human xenografts of NCI-H460 cells. In this study, the selected 2′-fluoropyrimidine RNA library was modified with PEG in order to increase its circulation time. After 11 rounds of selection, the aptamer RA16 demonstrated high affinity, binding selectively to NCI-H460 cells *in vivo*. In addition, RA16 showed functional activity by reducing the viability of NSCLC cells *in vitro* and tumor growth *in vivo* significantly.

It is well known that cancer induces changes in the invaded and adjacent tissue, creating a tumor microenvironment that favors tumor cell survival. Chen et al.^102^ performed *in vivo* SELEX to identify an aptamer for bone metastases in prostate cancer. A single-stranded DNA (ssDNA) thioaptamer library was injected in mice inoculated with prostate cancer cell line (PC3) that had metastasized in the bone (PB) and, after 10 rounds of selection, the PB aptamer, which bound to endothelial cells of metastatic bone lesions, was enriched. Surprisingly, *in vitro* assays showed that the PB aptamer was not recognizing tumor cells but, instead, an important component of the tumor microenvironment—activated endothelial cells. Further experiments were performed in endothelial cells (human umbilical vein endothelial cells [HUVECs]) conditioned with other prostate cancer cell lines, with results suggesting that aptamer binding was specific to the tumor microenvironment rather than a particular target cell or tissue.

Similarly, Haoran Liu et al.^103^ implanted MDA-MB-231 metastatic breast cancer cells in mice, and whole-organism *in vivo* SELEX was performed to enrich aptamers bound to the tumor as target tissue, and an MDA-MB-231 binding aptamer was identified. Surprisingly, the aptamer also showed high binding to polymorphonuclear myeloid-derived suppressor cells (MDSCs). MDSCs are highly infiltrating immunosuppressive immune cells that are abundant in the tumor milieu.

Likewise, Civit et al.^104^ implanted PC-3 prostate cancer tumor cells orthotopically in nude mice to perform whole-organism *in vivo* SELEX. As a novelty, two libraries were used to perform the SELEX, with one of them bearing PEG modification, which allows for increased half-life of aptamers *in vivo*. Success on aptamer selection was only achieved with the PEGylated library, putting into perspective that high kidney clearance of aptamers is still a major bottleneck for *in vivo* animal SELEX.

Whole-organism *in vivo* SELEX provides not only the possibility of targeting cancer cells, but also morphologically or structurally complex healthy tissues. For instance, Cheng et al.^105^ employed whole-organism *in vivo* SELEX and identified an aptamer capable of crossing the blood-brain barrier (BBB). Due to the complexity of the BBB structure, only *in vivo* SELEX provides an actual physiological choice to select aptamers that can access the brain. After 22 rounds of selection, the A15 aptamer was identified, which showed the ability to be internalized by endothelial cells of the BBB first, then penetrating the brain parenchyma. Moreover, data suggested higher affinity in parenchyma as compared to capillary cells after carotid artery injection.

#### Limitations of Whole-Organism *In Vivo* SELEX

One of the main caveats of whole-organism *in vivo* SELEX is that nucleic acids are susceptible to enzymatic degradation. The 2′-fluoropyrimidine modification is a pre-SELEX modification that significantly increases nuclease resistance, improving half-lives when administered in the bloodstream. Nonetheless, there might be aptamer species with higher content of 2′-fluoropyrimidines or with a structure that confers higher resistance to nucleases displaying higher longer half-lives in the bloodstream, thus introducing a bias in the selection.

Alternatively, when aptamers are injected systemically, elimination through the liver and clearance in the kidney are inevitable, which make the circulation half-life and time of harvest of target organs critical for SELEX success. In fact, studying the biodistribution of oligonucleotide aptamers after systemic injection shows higher abundance in the liver and kidney than in the other organs in spite of tissue specificity.^102^^,^^105^ This higher tropism to these two organs may likely reduce the complexity and diversity of species in the aptamer library, thereby reducing the chances of successful SELEX. Furthermore, there might be aptamer species that display structures more prone to liver or kidney elimination, skewing the selection. Conjugating the library with PEG increases the circulation time, suggesting that this modification enhances elimination of non-specific binders and might improve the whole-organism *in vivo* SELEX.^104^ Combining different variants of SELEX can also enhance the odds of success for *in vivo* selection. Along the early rounds of selection it might be desirable to start a simpler cell-SELEX against a particular tissue-specific cell line to gauge the restriction conditions of selection, and then as the selection progresses move into whole-organism *in vivo* SELEX to guarantee the *in vivo* functionality of aptamers selected.

### Conclusion

Aptamers, given their high specificity, versatile function, low immunogenicity, ability to be commercially manufactured in bulk in GMP grade, and relatively low cost of production, represent the next generation of therapeutics that deserve their spotlight in the clinic. However, a limitation to their therapeutic translation is their inability to function *in vivo* with the same efficacy as they do *in vitro*, especially when the aptamers are selected via conventional SELEX methods that utilize recombinant purified proteins as targets for the selection. With the advent of SELEX against live targets, that is, living cells, tissue samples, or even whole organisms, we have heralded a new era of aptamer discovery that ensures a higher translation potential to the clinic by allowing us to select aptamers that do not just simply bind to and modulate the target of choice, but do so in a physiologically appropriate and optimized manner, ensuring their therapeutic success *in vivo*, and subsequently in the clinic.

## Conflicts of Interest

The authors declare no competing interests.
